# The Adolescent Surgery Experience (ASE): a survey-based prospective cohort study to measure risk factors for persistent opioid use

**DOI:** 10.1016/j.bjao.2025.100496

**Published:** 2025-10-15

**Authors:** Tori N. Sutherland, Scott E. Hadland, Jiwon Moon, Joana Fardad, Elizabeth Ramsay, Michael J. Kallan, Mark D. Neuman

**Affiliations:** 1 Department of Anesthesiology and Critical Care, University of Pennsylvania Perelman School of Medicine, Philadelphia, PA, USA; 2 Leonard Davis Institute of Health Economics, University of Pennsylvania, Philadelphia, PA, USA; 3 Clinical Futures, Children’s Hospital of Philadelphia (CHOP), University of Pennsylvania Perelman School of Medicine, Philadelphia, PA, USA; 4 Division of Adolescent and Young Adult Medicine, Mass General for Children, Boston, MA, USA; 5 Department of Pediatrics, Harvard Medical School, Boston, MA, USA; 6 Center for Clinical Epidemiology and Biostatistics, University of Pennsylvania Perelman School of Medicine, Philadelphia, PA, USA; 7 Center for Perioperative Outcomes Research and Transformation, University of Pennsylvania Perelman School of Medicine, Philadelphia, PA, USA

**Keywords:** Adolescent Surgery Experience (ASE) study, new persistent opioid use, perioperative pain management, persistent postoperative pain, surgical recovery, risk factors for opioid use disorder (OUD)

## Abstract

**Background:**

Increasing data suggest adolescents have elevated risk of persistent postsurgical pain and opioid use, but their recovery experience remains poorly characterised.

**Methods:**

This prospective cohort study enrolled opioid-naive adolescents without chronic pain between June 2022 and May 2023 before undergoing procedures with anticipated mild, moderate, or severe postoperative pain. Participants completed eight surveys during recovery. We measured characteristics associated with persistent opioid use, including non-surgical site pain, difficulty sleeping, depression (Patient Health Questionnaire-9 [PHQ-9]), and anxiety (General Anxiety Disorder [GAD-7]) over 5 months after surgery.

**Results:**

Five hundred adolescents (median age: 15 yr (inter-quartile range 13–17 yr]) completed the baseline survey. Overall, 47.4% were female, 69.6% identified as White, 22.4% as Black/African American, and 10.8% as Hispanic/Latino. Overall, one in five (21.1%) reported depression, approximately two in five reported anxiety (37.4%), and one in six (16.6%) reported prior-year substance use. Among those undergoing procedures associated with severe pain, 93.4% received an outpatient opioid prescription (median 18 doses [inter-quartile range 12–25 doses]). At the end of the study, 16.7% (*n*=47) reported regular non-surgical site pain, 20.6% (*n*=58) had difficulty sleeping, and 15.7% and 15.3% had persistent depression and anxiety symptoms, respectively.

**Conclusion:**

A high proportion of adolescents endorsed preoperative anxiety, depression, and substance use, which, in combination with prescription opioids, are known risk factors for postoperative opioid use disorder. Over time, postoperative non-surgical site pain, difficulty sleeping, depression, and anxiety declined but remained common. Additional research is needed to understand the relationship between pre- and postoperative risk factors and adverse outcomes during surgical recovery.

**Clinical trial registration:**

NCT05482919.

Each year, >1.4 million US adolescents undergo surgery, and there is growing concern among experts that they are at increased risk of adverse outcomes related to opioid use and chronic pain.^1^^,^^2^ New persistent opioid use; using opioid prescription refills 3–6 months after surgery as a proxy,^3^ may represent a new opioid use disorder (OUD). It has been identified in 3.0–4.8% of previously opioid-naive adolescents undergoing surgery.^2^^,^^4^

Conditions associated with persistent opioid use include non-surgical chronic pain, substance use disorders, insomnia, attention deficit hyperactivity disorder (ADHD), and mental health diagnoses.^2^^,^^3^^,^^5^, ^6^, ^7^, ^8^, ^9^ Prescription factors, specifically total duration (days) of opioid exposure, preoperative dispensing, and high daily dose dispensed in relation to anticipated pain severity are also associated.^4^^,^^10^^,^^11^ New chronic pain after major surgery affects approximately 20% of paediatric patients, suggesting that while opioid use may reflect development of an OUD, it may also be related to attempts to treat persistent postsurgical pain.^12^, ^13^, ^14^ Existing studies on postsurgical persistent pain include a higher proportion of younger adolescents, despite concerns of increased adverse outcomes among older adolescents and other underrepresented groups.^4^^,^^15^

The trajectory from surgery to development of these adverse outcomes remains poorly characterised in the context of individual characteristics and perioperative pain management. A better understanding of the adolescent experience will assist researchers and clinicians to characterise individual risk factors and design effective interventions to prevent adverse outcomes. Here, we describe our study procedures and our baseline cohort, including perioperative pain management and characteristics associated with persistent opioid use up to 5 months after surgery.

## Methods

### Design, setting, and participants

We conducted a prospective observational cohort study at a quaternary paediatric hospital system and followed the Strengthening the Reporting of Observational Studies in Epidemiology (STROBE) guidelines.^16^ Adolescents were enrolled between June 2022 and May 2023 before undergoing procedures associated with mild, moderate, and severe postoperative pain and observational data was collected for 5 months after surgery. The study was approved by the Children’s Hospital of Philadelphia (CHOP) institutional review board and registered with ClinicalTrials.gov (NCT05482919).

Enrolment occurred at CHOP’s quaternary facility in Philadelphia, PA and at a tertiary hospital in King of Prussia, PA, an adjacent suburban community; approximately 40 000 anaesthetics were performed each year across the hospital system. Eligible patients included adolescents aged 11–21 yr with an American Society of Anesthesiologists (ASA) physical status ≤3 who were scheduled for noncardiac surgery. Exclusion criteria included lack of informed consent/assent, concern that patients/caregivers were unable to provide informed consent, fully understand study procedures, or both, participants with limited English proficiency and caregivers with limited proficiency if a certified interpreter was not available, history of pre-existing chronic pain (pain present for >3 months), and self-reported lifetime prescription opioid use >5 consecutive days for acute pain or >10 days for major surgery. After screening, patients were approached by a study team member on the day of surgery or by phone before surgery to discuss the study, answer questions, and obtain consent if patients and caregivers chose to enrol. Informed consent was obtained from caregivers and adolescents aged 18–21 yr and assent from those aged 11–17 yr using a combined consent/HIPAA authorisation form hosted on CHOP’s REDCap (Research Electronic Data Capture) platform.^17^ If a patient reached 18 yr during the study period, a new informed consent was completed by phone and signed remotely using REDCap.

### Characteristics associated with high-risk opioid use

Herein, we present the prevalence of characteristics associated with high-risk opioid use up to 5 months after surgery, including regular non-surgical site pain, difficulty sleeping, and anxiety and depression symptoms measured by validated instruments. Because incidence is elevated after major procedures, such as spinal fusion, we captured self-reported persistent surgical site pain among all patients and among those undergoing severe pain procedures.

#### Data collection

**Fig 1:**
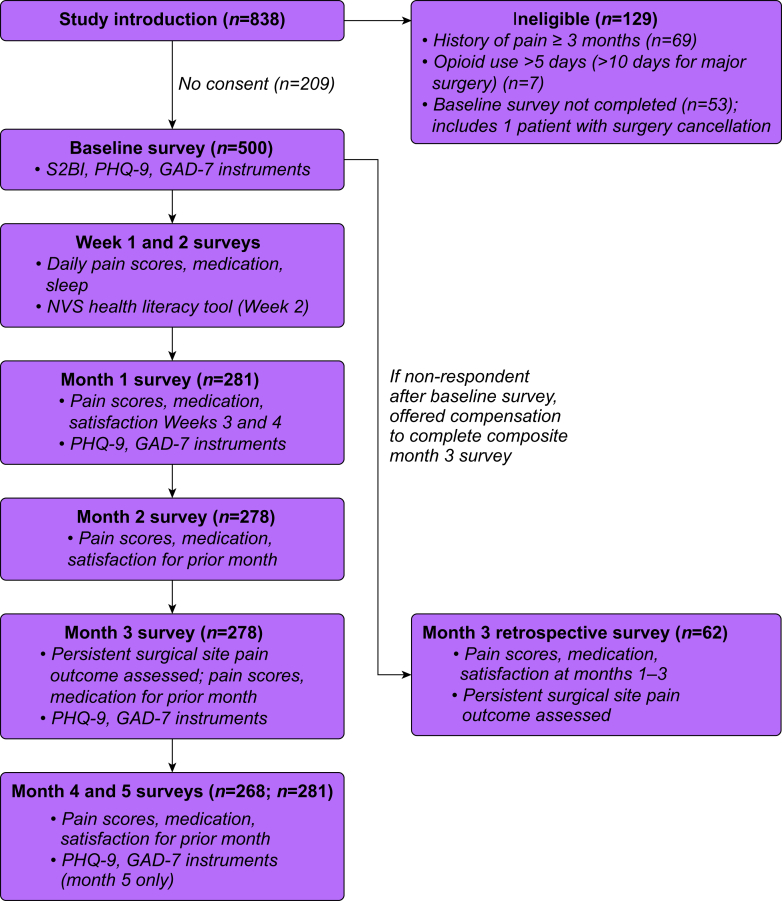
Overview of study procedures. GAD-7, Generalized Anxiety Disorder-7; NVS, Newest Vital Sign; PHQ-9, Patient Health Questionnaire-9.

Participants completed eight surveys during the study period at the following intervals: baseline 2 weeks up to day of surgery, week 1, week 2, and months 1–5 (Figure 1). Surveys were hosted on the REDCap platform and sent to the adolescent’s smartphone embedded within a text message link. Participants also had the option of completing surveys by phone call or e-mail, although none selected these options. The baseline survey contained screening questions to verify patients did not have substantial prior opioid exposure, which was previously defined, or a history of pre-existing pain for >3 months. The latter question was revised to emphasise the duration of pain after multiple adolescents failed screening during the pilot phase and expressed they did not understand the question. The survey also contained general demographic and baseline characteristic questions, including participant and caregiver educational attainment, languages spoken at home, race/ethnicity, family members with chronic pain, sleep schedule, and history of anxiety, depression, and ADHD. The Screening to Brief Intervention (S2BI) instrument was used to screen for substance use.^18^ Adolescents also completed the Patient Health Questionnaire-9 (PHQ-9) and Generalized Anxiety Disorder-7 (GAD-7) questionnaires with the baseline, month 1, 3, and 5 surveys to measure depression and anxiety symptoms, respectively.^19^^,^^20^ One patient experienced a cancellation on the day of surgery, went on to complete a postoperative survey, and was excluded from the final analysis. Two research assistants collected data from the patient’s electronic medical record (EMR) including the final surgical case booking, medical co-morbidities, and all medications administered in the hospital or prescribed for pain management at home. These data were abstracted together by a two-person team and approximately 10% of forms were randomly selected for audit to ensure data accuracy.

The week 1 and week 2 surveys were distributed as a handout that could be uploaded to the database as a screenshot or filled out in REDCap. Data included daily medication doses, average and maximum pain scores on a 0–10 scale, average hours of sleep, percentage of each day in severe pain, surgical site pain quality, any non-surgical site pain, and satisfaction with recovery on a five-point scale. If a participant was inpatient, the study research coordinators entered medication doses and pain scores documented in the EMR. Adolescents also completed Pfizer’s Newest Vital Sign (NVS) questionnaire to assess health literacy in the week 2 survey.^21^ The months 1–5 surveys contained questions about pain experience and pain medication use (weeks 3–4 for the month 1 survey).

#### Retention and retrospective Month 3 survey

After completion of 6 weeks of pilot data collection beginning in June 2022, we made several changes to improve retention. These included moving the health literacy questionnaire from the baseline survey to the week 2 survery as staff noted that patients were often unable to complete this before surgery. In addition, we began to contact patients up to 1 week before a scheduled procedure to allow additional time to complete the baseline survey. We re-distributed compensation to encourage completion of the month 3 survey where we measured the persistent surgical site pain outcome. Mid-study, we also began to send two personal text messages containing survey links to adolescents who had been non-respondent for >3 days for select surveys.

For patients who were non-respondent after the baseline survey despite these measures, we also obtained institutional review board (IRB) approval to send a retrospective month 3 survey to participants undergoing moderate and severe pain procedures who would have otherwise been lost to follow-up. This survey asked about opioid use after surgery between months 1 and 3, persistent pain at month 3, and overall satisfaction with recovery. We also compared characteristics of all patients and those who provided information on the persistent pain outcome at month 3.

#### Surgical procedure classification

Procedures were categorised according to anticipated level of postoperative pain by two experienced pain management specialists. Because the primary objective was to better understand an adolescent’s recovery trajectory, we included procedures associated with mild and moderate pain as they are frequently associated with persistent opioid use.^4^ Study procedures included those associated with mild pain (wisdom teeth extraction, endoscopy), moderate pain (laparoscopic cholecystectomy, knee arthroscopy without ligament repair, tonsillectomy, mammoplasty), and severe pain (spinal fusion for scoliosis, knee arthroscopy with ligament repair, nuss bar insertion). If this assignment was uncertain, the operative note was reviewed by the primary investigator and a senior pain management team provider with regard to extent of noxious injury and typical postoperative pain levels.

### Statistical analysis

#### Sample size calculations

We measured persistent surgical site pain at month 3 among all patients and those undergoing procedures associated with severe pain. We planned to enrol 300 patients after reviewing case volume and logistical constraints. Because persistent pain was poorly characterised but anticipated to be higher among patients undergoing severe pain procedures, the study was powered (0.90; significance level 0.05) to detect a difference between the historic chronic pain incidence of 20% and a lower chronic pain incidence of 11%^22^ after undergoing general and orthopaedic procedures associated with severe postoperative pain. We planned to enrol a minimum of 150 patients undergoing procedures associated with severe pain to allow for up to 20% dropout (goal: 115 patients with data at month 3) and to enrol the remainder of participants who were scheduled for mild and moderate pain procedures as these are also associated with adverse postsurgical outcomes, including new persistent opioid use.

#### Data analysis

We compared patient characteristics and potential exposures, including preoperative anxiety, depression, and substance use, among the three procedural pain groups. We also compared demographic and exposure measures among patients who completed the baseline survey *vs* those who provided data on persistent surgical pain at month 3. Data were analysed in SAS software Version 9.4 (SAS Institute Inc, Cary, NC, USA). If a comparative test was appropriate, tests were two-sided, and significance was set at 5%.

## Results

The study cohort included 500 opioid-naive adolescents without chronic pain who completed the preoperative baseline survey (Table 1). Their median age was 15 yr (inter-quartile range [IQR] 13–17 yr), and 47.4% were female. With regard to race and ethnicity, 22.4% reported their race as black/African American, 69.6% as white, and 10.8% identified as having Hispanic/Latino ethnicity. As reference, 56.4% of adolescents receiving care at CHOP who reported their race/ethnicity identified as an underrepresented group, and 25.6% identified as black/African American. Approximately one-third of caregivers completed some high school or received a high school degree as their highest educational attainment, and 18.0% reported that a caregiver or close family member had a history of chronic pain. Among those who completed the health literacy assessment (*n*=358), 6.7% likely had limited literacy, and 15.4% possibly had limited literacy. These proportions were not substantially different among the adolescents who also provided data on the primary outcome at month 3 (*n*=340; Supplementary Table S1) or among the three procedural pain categories.

**Table 1:** Patient characteristics, baseline completed (*n*=500). IQR, inter-quartile range. ∗No response for one participant in mild and moderate pain groups.

Patient characteristics	Mild pain procedures (*n*=64)	Moderate pain procedures (*n*=254)	Severe pain procedures (*n*=182)	All procedures (*n*=500)
Age (yr), median (IQR)	15 (13.5–17)	15 (13–17)	15 (13–17)	15 (13–17)
Female sex, *n* (%)	29 (45.3)	113 (44.5)	95 (52.2)	237 (47.4)
Race/ethnicity, *n* (%)				
White	42 (65.6)	174 (68.5)	132 (72.5)	348 (69.6)
Black/African American	14 (21.9)	59 (23.2)	39 (21.4)	112 (22.4)
American Indian or Alaska Native	1 (1.6)	3 (1.2)	3 (1.6)	7 (1.4)
Asian	5 (7.8)	14 (5.5)	7 (3.8)	26 (5.2)
Native Hawaiian or Pacific Islander	0 (0.0)	3 (1.2)	1 (0.5)	4 (0.8)
Multiracial	1 (1.6)	0 (0.0)	0 (0.0)	1 (0.2)
Unknown	1 (1.6)	1 (0.4)	0 (0.0)	2 (0.4)
Hispanic/Latino∗	8 (12.7)	32 (12.6)	14 (7.7)	54 (10.8)
Type of school attended, *n* (%)∗				
Elementary	1 (1.6)	7 (2.8)	5 (2.7)	13 (2.6)
Middle school	16 (25.4)	66 (26.1)	50 (27.5)	132 (26.5)
High school	39 (61.9)	141 (55.7)	112 (61.5)	292 (58.6)
College/technical	5 (7.9)	28 (11.1)	11 (6.0)	44 (8.8)
Not in school	2 (3.2)	11 (4.3)	4 (2.2)	17 (3.4)
Speaks non-English language at home, *n* (%)	7 (10.9)	17 (6.7)	7 (3.8)	31 (6.2)
Caregiver educational attainment, *n* (%)∗				
Did not complete high school	4 (6.3)	9 (3.6)	5 (2.7)	18 (3.6)
High school	16 (25.4)	87 (34.4)	60 (33.0)	163 (32.7)
College	27 (42.9)	94 (37.2)	68 (37.4)	189 (38.0)
Graduate school	8 (12.7)	35 (13.8)	34 (18.7)	77 (15.5)
Unsure	6 (9.5)	18 (7.1)	7 (3.8)	31 (6.2)
Prefer not to answer	2 (3.2)	10 (4.0)	8 (4.4)	20 (4.0)
Family member with chronic pain, *n* (%)				
Parent/primary caregiver	10 (15.6)	33 (13.0)	32 (17.5)	75 (15.0)
Other family members in home	4 (6.3)	8 (3.1)	3 (1.6)	15 (3.0)
Sleep				
Median number hours sleep, (IQR)	8 (7-9)	8 (8-9)	8 (7-9)	8 (7-9)
Newest Vital Sign (NVS) health literacy score (*N*=358)	(*n*=42)	(*n*=183)	(*n*=133)	(*n*=358)
Limited literacy likely (0–1)	2 (4.8)	13 (7.1)	9 (6.8)	24 (6.7)
Possible limited literacy (2–3)	8 (19.0)	33 (18.0)	14 (10.5)	55 (15.4)
Likely adequate literacy (4–6)	32 (76.2)	137 (74.9)	110 (82.7)	279 (77.9)

Table 2 displays characteristics associated with increased risk of opioid-related adverse events. Overall, 14.9% had an ADHD diagnosis and 30.7% of adolescents had difficulty sleeping one or more nights each week at baseline. In the USA, ∼11.4% paediatric patients have an ADHD diagnosis and nearly 11% have been diagnosed with insomnia, although a higher proportion likely experience difficulty sleeping.^23^^,^^24^ Among the 21.1% who reported a history of depression, 29.2% had preoperative symptoms consistent with mild, moderate, or severe depression on the PHQ-9 assessment. Among the 37.3% who reported a history of anxiety, 22.0% had clinically significant anxiety symptoms before surgery on the GAD-7 assessment. Regarding substance use in the previous year, 16.6% of youths most commonly endorsed alcohol use (13.2%) and marijuana use (9.2%). Among those consuming marijuana and vape products, both groups reported a median 10 days (IQR 2–100 days) of use.

**Table 2:** Baseline characteristics associated with development of persistent opioid use (*n*=500). GAD-7, Generalized Anxiety Disorder-7 (screening tool for anxiety); PHQ-9, Patient Health Questionnaire-9 (screening tool for depression); S2BI, Screening to Brief Intervention (screening tool for substance use). ∗No response for one participant in mild and moderate pain groups.

Patient characteristics	Mild pain procedures (*n*=64)	Moderate pain procedures (*n*=254)	Severe pain procedures (*n*=182)	All procedures (*n*=500)
ADHD diagnosis, *n* (%)∗	8 (12.7)	41 (16.2)	25 (13.7)	74 (14.9)
History of difficulty sleeping 1+ episodes/week, *n* (%)∗	24 (38.1)	74 (29.2)	55 (30.2)	153 (30.7)
History of depression, *n* (%)∗	13 (20.6)	60 (23.7)	32 (17.6)	105 (21.1)
PHQ-9 score				
0–4	43 (67.2)	182 (71.7)	129 (70.9)	354 (70.8)
5–9	14 (21.9)	52 (20.5)	37 (20.3)	103 (20.6)
10–14	5 (7.8)	15 (5.9)	13 (7.1)	33 (6.6)
15–19	2 (3.1)	2 (0.8)	1 (0.5)	5 (1.0)
20+	0 (0.0)	3 (1.2)	2 (1.1)	5 (1.0)
History of anxiety, *n* (%)∗	26 (41.3)	90 (35.6)	70 (38.5)	186 (37.3)
GAD-7 score∗, *n* (%)				
0–4	47 (73.4)	197 (77.6)	146 (80.2)	390 (78.0)
5–9	6 (9.4)	40 (15.7)	20 (11.0)	66 (13.2)
10–14	4 (6.3)	9 (3.5)	13 (7.1)	26 (5.2)
15–21	7 (10.9)	8 (3.1)	3 (1.6)	18 (3.6)
If problems (score >0), how difficult is life:	(*n*=36)	(*n*=157)	(*n*=118)	(*n*=311)
Not difficult at all	19 (52.8)	117 (74.5)	77 (65.3)	213 (68.5)
Somewhat difficult	11 (30.6)	29 (18.5)	33 (28.0)	73 (23.5)
Very difficult	5 (13.9)	8 (5.1)	7 (5.9)	20 (6.4)
Extremely difficult	1 (2.8)	3 (1.9)	1 (0.8)	5 (1.6)
S2BI score∗; *n* (%) endorsed substance use in past year				
Cigarettes/tobacco	2 (3.1)	10 (3.9)	5 (2.7)	17 (3.4)
Alcohol	9 (14.1)	38 (15.0)	19 (10.4)	66 (13.2)
Marijuana	5 (7.8)	23 (9.1)	18 (9.9)	46 (9.2)
Vape	4 (6.3)	16 (6.3)	15 (8.2)	35 (7.0)
Among those who endorsed use, median days use (IQR)				
Cigarettes/tobacco	1 (1–1)	6.5 (2–10)	10 (2–20)	5 (1–10)
Alcohol	1 (1–5)	10 (3–30)	10 (3–20)	10 (2–25)
Marijuana	5 (2–10)	20 (3–150)	4 (2–75)	10 (2–100)
Vape	2.5 (1–4.5)	30 (4.5–160)	20 (2–100)	10 (2–100)

Supplementary Table S2 displays aspects of the adolescent’s surgical experience on the day of surgery, including the most common procedures, admission status, and intraoperative pain treatment. During surgery, pain management was multi-modal: 83.8% received at least one opioid dose, 52.4% received ketorolac, 56.6% received acetaminophen, and 31.2% had either a single-shot or continuous-catheter nerve block. By procedural pain category, Supplementary Table S3 summarises medication prescribed for pain management at home. Overall, 61.4% received an opioid prescription for use after surgery, and the proportion increased with the severity of anticipated postoperative pain. Among patients undergoing a procedure associated with severe postoperative pain, the median prescription contained 18 doses (IQR 12–25 doses).

**Fig 2:**
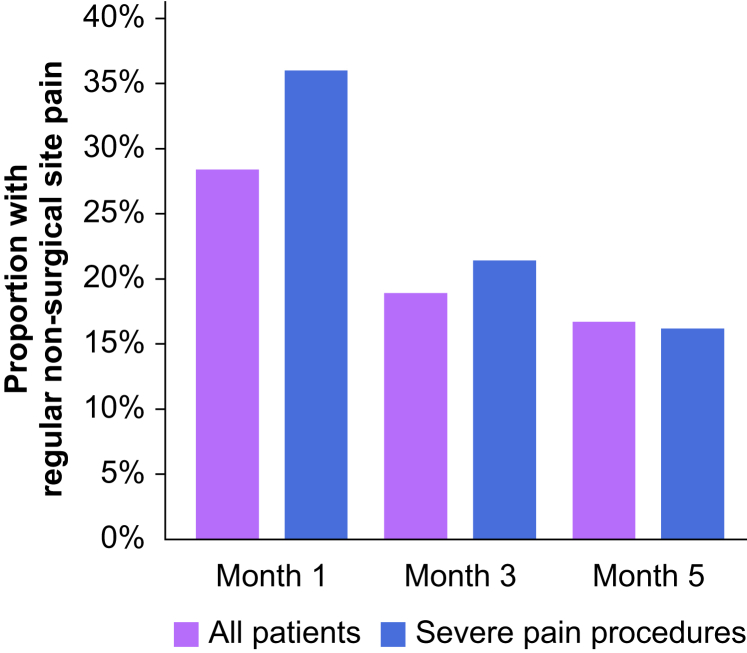
Regular non-surgical site pain in postoperative Months 1, 3, and 5, overall and severe pain procedures only.

**Fig 3:**
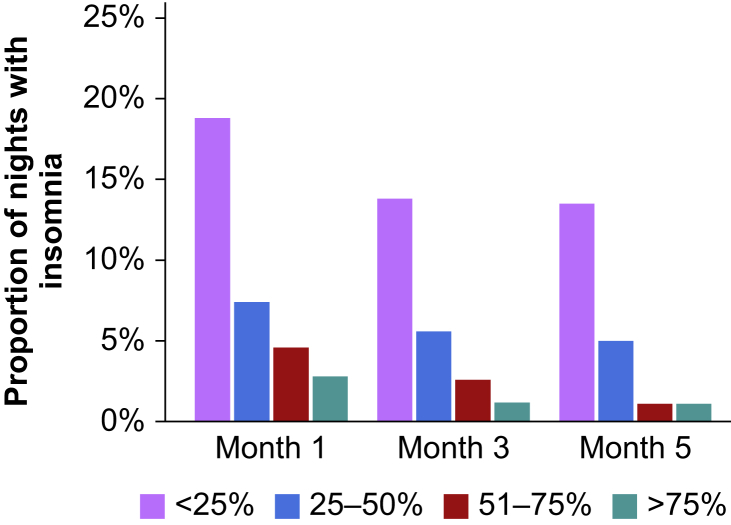
Proportion of nights with difficulty sleeping in postoperative Months 1, 3, and 5 among those with insomnia, all patients.

We also captured the prevalence of regular non-surgical site pain, difficulty sleeping, PHQ-9, and GAD-7 scores between postoperative months 1 and 5, by all patients and among those recovering from severe pain procedures (Supplementary Table S4, Fig 2, Fig 3). Between months 1 and 5, non-surgical site pain declined from 28.4% to 16.7% overall, with headache being most commonly reported and the most substantial decline between month 1 and month 2. A slightly higher proportion reported pain in month 1 (36.0%) after severe pain procedures, although a similar decline occurred by month 5 (16.2%), with the majority of the decrease related to back pain. Overall, 14.8% (*n*=42) reported difficulty sleeping more than 1 week in month 1 compared with 7.2% (*n*=20) in month 5 with a decrease in the proportion reporting difficulty sleeping >50% of nights. There was also a decline in the proportion reporting clinical symptoms of depression (PHQ-9 score >4; 21.6% *vs* 15.7%) between months 1 and 5, although 3.5% (*n*=10) experienced moderate to severe symptoms in month 5. Both the proportion with clinical anxiety symptoms and symptom severity remained relatively stable between months 1 and 5.

## Discussion

In this cohort study, we enrolled 500 adolescents to report on baseline characteristics, perioperative pain management and measured characteristics associated with development of persistent opioid use up to 5 months after surgery. Participants were diverse and included older adolescents (15–21 yr) that are often underrepresented in prospective surgical outcomes research. A high proportion endorsed regular non-surgical site pain, difficulty sleeping, anxiety, and depression, which are associated with developing opioid use discorder (OUD) after surgery. The incidences of pain, difficulty sleeping, and depression declined during the study period, while anxiety symptoms remained stable. However, at the end of the study, one in six adolescents described regular non-surgical site pain, and one in five reported persistent difficulty sleeping. It was also concerning that one in six endorsed substance use in the prior year, as use of cannabis, alcohol, and nicotine in adolescence is associated with adverse consequences ranging from poor mental health to elevated risk of future substance use disorders.^25^

It is important to consider these findings in the context of significant increases in the global prevalence of anxiety and depression after 2020 during the period of study enrollment.^26^ It is possible that the prevalence of mental health conditions identified in this study may reflect regional trends. It is also unclear why depression, but not anxiety, scores declined between months 1 and 5. Regardless, these findings highlight the high prevalence of risk factors for persistent opioid use among adolescents recovering from surgery. Future research is indicated to better understand the recovery experience, including impact of non-surgical site pain and difficulty sleeping and relationship with postoperative adverse outcomes. Additional outcomes that will be analysed after completion of data collection include postsurgical persistent pain, using the International Association for the Study of Pain (IASP) definition of chronic pain that is self-reported, develops or increases in intensity at the surgical site, and is present >3 months after surgery.^27^ We will also measure opioid use in weeks 3 and 4, time in days to resolution of severe pain (self-reported; <7/10 daily average), and satisfaction with recovery.

When designing future studies, our experiences offer valuable lessons for perioperative outcomes researchers who work with adolescents. During the data collection process, we prioritised diversity in enrollment, retention, and data completeness. We collaborated closely with surgeons who allowed our group unrestricted access to approach their patients. We focused on select procedures and enrolling a cohort that was representative of racial and ethnic diversity at the study site. Although we experienced loss to follow-up after the baseline survey, we implemented several retention measures, including text message follow-up and compensation that strategically increased for surveys where outcomes were measured (month 3). We also obtained IRB approval to contact participants who were non-respondent after the baseline survey to complete a retrospective month 3 survey to capture composite outcomes data. We were uncertain if outcomes would differ among these adolescents and planned to examine this after study completion. We were also reassured that those who provided baseline data only and those who also provided outcome data at month 3, including those who completed the retrospective month 3 survey, had similar baseline characteristics. The level of engagement, particularly among adolescents undergoing more invasive procedures, was notable and a reminder of the importance of engaging adolescents in their medical care as they progress towards independence. As an example, retention was close to 100% with shorter surveys between months 1 and 5, with compensation aligned strategically with survey completion. We also began to follow up mid-study with two personal text messages containing survey links. These findings also suggest that when possible, studies involving adolescents enrolled in school should limit data collection to surveys that require <5 min to complete.

Opioid prescriptions, as part of a multi-modal regimen, were dispensed with a smaller number of doses compared with national averages for similar procedures, and refill prescriptions are rare at the study hospitals.^4^ After undergoing procedures associated with severe pain, adolescents filled an initial prescription containing an average of 18 opioid doses. In contrast, an initial opioid prescription dispensed to a US adolescent undergoing major surgery contained an average of 59 hydrocodone 5 mg tablets, and nearly one in five filled a second prescription within 30 days of surgery that contained an average 45 hydrocodone 5 mg tablets.^4^ A recent prospective study on opioid consumption among adolescents concluded that an improved understanding of patient and procedural factors impacting opioid use was necessary to reduce excess opioid dispensing.^28^

Adolescents also commonly filled prescriptions for benzodiazepines for musculoskeletal procedures. Co-prescribing of opioids with benzodiazepines is often not recommended because of concerns of excess sedation, respiratory depression, or both, and these prescriptions were generally limited to procedures for which the benefit of a limited course may outweigh the risks. Providers at the study institution routinely counsel patients on these risks, and after the study period, they began to co-prescribe naloxone with all opioid prescriptions.

The primary limitation of this analysis relates to data collection at a single site and possible lack of generalisability. However, our patient population was diverse with regard to socioeconomic status, using caregiver educational attainment as a proxy, and race/ethnicity. There was increased representation of adolescents over 15 yr compared with existing literature, which is important because opioid-related adverse outcomes, including exposures associated with development of OUD, begin to increase at this age.^10^ Because recall bias was also possible, we structured surveys to focus on experiences in the days to weeks immediately before surveys. We also experienced loss to follow-up before measurement of persistent surgical site pain at month 3. It was reassuring that patient characteristics among those who provided data at Month 3 were similar to the full baseline cohort.

In conclusion, this study provided valuable insight on the prevalence of risk factors for persistent opioid use over time. Findings also provide guidance for study design to effectively recruit and retain a diverse cohort of adolescents undergoing surgery. This cohort reported a high prevalence of characteristics associated with opioid-related adverse outcomes, including regular non-surgical site pain, difficulty sleeping, depression, anxiety, and prior substance use. Although there was a decline in pain, difficulty sleeping, and depression over time, the relationship between these factors and development of high-risk opioid use remains poorly characterised, and future research is indicated.

## Authors’ contributions

Study conception: TNS, SEH, MDN

Study design: TNS, SEH, MDN

Data analyses: MJK, TNS, JM, JF, EB

Data interpretation: all authors

Drafting first and final version of manuscript: TNS, SHE, MDN

Critically revising work for important intellectual content and approving final version: all authors

## Ethics approval statement

This study was approved by the Children’s Hospital of Philadelphia Institutional Review Board and registered with ClinicalTrials.gov (NCT05482919). Consent (parent and adolescents ≥18 yr) and assent when appropriate under age 18 was obtained before enrollment.

## Funding

A Foundation for Anesthesia Education and Research (FAER) MRTG award (Dr. Sutherland). The FAER had no role in the design and conduct of the study.

## Declarations of interest

SEH reported honoraria related to speaking on substance use from the American Academy of Pediatrics outside the submitted work. No other disclosures were reported.
