# Supplementary material for: The Adolescent Surgery Experience (ASE): a survey-based prospective cohort study to measure risk factors for persistent opioid use
Source: BJA Open. 2025 Oct 15;16:100496. doi: 10.1016/j.bjao.2025.100496 (PMC12553083; doi:10.1016/j.bjao.2025.100496)
Supplement: Multimedia component 1 [file mmc1.docx]

| **Appendix Table 1: Patient demographics, Baseline and Month 3 surveys completed (n=340)** | | | | |
| --- | --- | --- | --- | --- |
| **Patient characteristics** | **Mild** (n=33) | **Moderate** (n=166) | **Severe** (n=141) | **All** (n=340) |
| Age, median (IQR) | 15 (14-17) | 15 (14-17) | 15 (13-16) | 15 (13.5-17) |
| Female gender, n(%) | 16 (48.5) | 74 (44.6) | 77 (54.6) | 167 (49.1) |
| **Race/Ethnicity, n(%)** | - | - | - | - |
| White  Black/African American  American Indian or Alaska Native  Asian  Native Hawaiian or Pacific Islander  Multiracial  Unknown | 23 (69.7)  6 (18.2)  1 (3.0)  3 (9.1)  0 (0.0)  0 (0.0)  0 (0.0) | 117 (70.5)  36 (21.7)  1 (0.6)  11 (6.6)  1 (0.6)  0 (0.0)  0 (0.0) | 105 (74.5)  27 (19.1)  3 (2.1)  5 (3.5)  1 (0.7)  0 (0.0)  0 (0.0) | 245 (72.1)  69 (20.3)  5 (1.5)  19 (5.6)  2 (0.6)  0 (0.0)  0 (0.0) |
| Hispanic/Latino | 6 (18.2) | 18 (10.8) | 11 (7.8) | 35 (10.3) |
| **Type of school attended, n(%)**  Elementary  Middle school  High school  College/Technical  Not in school | 0 (0.0)  8 (24.2)  22 (66.7)  3 (9.1)  0 (0.0) | 2 (1.2)  43 (25.9)  97 (58.4)  16 (9.6)  8 (4.8) | 4 (2.8)  42 (29.8)  83 (58.9)  8 (5.7)  4 (2.8) | 6 (1.8)  93 (27.4)  202 (59.4)  27 (7.9)  12 (3.5) |
| **Speaks non-English language at home, n(%)** | 4 (12.1) | 8 (4.8) | 3 (2.1) | 15 (4.4) |
| **Primary caregiver education level, n(%)**  Did not complete high school  High school  College  Graduate school  Unsure  Prefer not to answer | 1 (3.0)  8 (24.2)  16 (48.5)  2 (6.1)  5 (15.2)  1 (3.0) | 6 (3.6)  57 (34.3)  63 (38.0)  24 (14.5)  11 (6.6)  5 (3.0) | 4 (2.8)  42 (29.8)  56 (39.7)  26 (18.4)  6 (4.3)  7 (5.0) | 11 (3.2)  107 (31.5)  135 (39.7)  52 (15.3)  22 (6.5)  13 (3.8) |
| **Family member with chronic pain, n(%)**  Parent/primary caregiver  Other family members in home | 5 (15.2)  1 (3.0) | 20 (12.0)  5 (3.0) | 23 (16.3)  3 (2.1) | 48 (14.1)  9 (2.6) |
| **Sleep**  Median number hours sleep (IQR) | 8 (7-9) | 8 (8-9) | 8 (7-9) | 8 (7-9) |
| **History of difficulty sleeping** 1+ episodes/week, n(%) | 11 (33.3) | 47 (28.3) | 49 (34.8) | 107 (31.5) |
| **History of depression**, n(%) | 7 (21.2) | 31 (18.7) | 25 (17.7) | 63 (18.5) |
| **History of anxiety**, n(%) | 15 (45.5) | 57 (34.3) | 56 (39.7) | 128 (37.6) |
| **ADHD diagnosis**, n(%) | 5 (15.2) | 31 (18.7) | 22 (15.6) | 58 (17.1) |
| **S2BI score**†**; n(%) who endorsed use**  Cigarettes/tobacco  Alcohol  Marijuana  Vape  **Among those who endorsed use, median days use (IQR)**  Cigarettes/tobacco  Alcohol  Marijuana  Vape | 2 (6.1)  8 (24.2)  4 (12.1)  3 (9.1)  1 (1-1)  1.5 (1-15)  3.5 (1.5-185)  1 (1-5) | 5 (3.0)  18 (10.8)  11 (6.6)  5 (3.0)  8 (5-10)  10 (2-30)  20 (4-150)  50 (40-200) | 2 (1.4)  13 (9.2)  11 (7.8)  10 (7.1)  76 (2-150)  10 (3-15)  3 (1-75)  22.5 (3-100) | 9 (2.6)  39 (11.5)  26 (7.6)  18 (5.3)  5 (1-10)  5 (2-25)  12.5 (2-75)  22.5 (2-100) |
| †Abbreviation: Screening to Brief Intervention (S2BI; screening tool for substance use) | | | | |

| **Appendix Table 2. Characteristics of perioperative experience, All patients (n=500)** | | | | |
| --- | --- | --- | --- | --- |
|  | **Mild** (n=64) | **Moderate** (n=254) | **Severe** (n=182) | **All** (n=500) |
| Common procedures | Dental Extraction (27)  Endoscopy (18)  Cystoscopy (5) | T&A (33)  Mammoplasty (22)  Knee Arthroscopy without Ligament Involvement (20) | Knee Arthroscopy with Ligament Involvement (82)  Spinal Fusion (38)  Nuss Bar Insertion (14) | Knee Arthroscopy with Ligament Involvement (82)  Spinal Fusion (38)  T&A (33) |
| Inpatient admission, n(%)  Stay, in days, median (IQR) | 2 (3.1)  1 (1-1) | 23 (9.1)  2 (1-3) | 26 (14.3)  2.5 (2-3) | 51 (10.2)  2 (1-3) |
| **Preoperative medications** |  |  |  |  |
| Oral midazolam, n(%) | 37 (58.7) | 171 (67.6) | 124 (68.1) | 332 (66.7) |
| Acetaminophen n(%) | 0 (0.0) | 2 (0.8) | 2 (1.1) | 4 (0.8) |
| **Intra-operative medications** |  |  |  |  |
| Opioid, n(%)  *Fentanyl*  *Hydromorphone*  *Methadone*  *Morphine*  Average dose quantity (sd)  Fentanyl, ucg  Hydromorphone, mg  Methadone, mg  Morphine, mg | 48 (75.0)  *33*  *4*  *0*  *17*  78.0 (32.3)  0.5 (0.1)  -  2.6 (1.3) | 233 (91.7)  *134*  *77*  *0*  *102*  106.8 (58.3)  1.6 (6.8)  -  4.1 (2.0) | 138 (75.8)  *107*  *31*  *52*  *36*  145.3 (113.1)  0.8 (0.5)  6.2 (2.2)  3.0 (1.5) | 419 (83.8)  *274*  *112*  *52*  *155*  118.3 (85.4)  1.3 (5.6)  6.2 (2.2)  3.7 (1.9) |
| Ketorolac, n(%)  Avg dose/kg, mean(sd) | 26 (40.6)  22.8 (7.0) | 119 (46.9)  23.6 (6.8) | 117 (43.3)  25.4 (6.3) | 262 (52.4)  24.4 (6.7) |
| Acetaminophen, n(%)  Avg dose/kg, mean(sd) | 12 (18.8)  774.6 (278.7) | 123 (48.4)  890.4 (176.6) | 148 (81.3)  866.7 (180.0) | 283 (56.6)  873.1 (184.3) |
| Ketamine, n(%)  Avg dose/kg, mean(sd) | 0 (0.0)  - | 1 (0.4)  200.0(-) | 10 (5.5)  51.8 (23.5) | 11 (2.2)  65.3 (49.9) |
| Regional adjuvant, n(%)  *Single-shot nerve block*  *Continuous catheter* | 0 (0.0)  *0*  *0* | 47 (18.5)  *45*  *5* | 109 (59.9)  *109*  *13* | 156 (31.2)  *154*  *18* |

| **Appendix Table 3. Medications prescribed for home, all patients** | | | | |
| --- | --- | --- | --- | --- |
|  | **Mild** (n=64) | **Moderate** (n=254) | **Severe** (n=182) | **All** (n=500) |
| Opioid, n(%)  *Hydromorphone*  *Morphine*  *Oxycodone*  Days prescribed, median (IQR)  Doses prescribed, median (IQR) | 4 (6.3) [n=63]  *0*  *0*  *4*  1.5 (1-2.5)  6 (4.5-12) | 132 (52.2) [n=253]  *0*  *0*  *132*  3 (2-3)  12 (10-17) | 170 (93.4)  *3*  *1*  *166*  4 (3-5)  18 (12-25) | 306 (61.4) [n=498]  *3*  *1*  *302*  3 (2-4)  15 (12-20) |
| Benzodiazepine, n(%) | 1 (1.6) | 18 (7.1) | 80 (44.0) | 99 (19.8) |
| NSAID, n(%) | 28 (43.8) | 166 (65.4) | 164 (90.1) | 358 (71.6) |
| Acetaminophen, n(%) | 34 (53.1) | 232 (91.3) | 179 (98.4) | 445 (89.0) |
| Gabapentin/pregabalin, n(%) | 1 (1.6) | 3 (1.2) | 1 (0.5) | 5 (1.0) |

| **Appendix Table 4. Non-surgical site pain, difficulty sleeping and mental health symptoms, Months 1-5** | | | | | |
| --- | --- | --- | --- | --- | --- |
| **Overall** | | | | | |
|  | **Month 1**  **(n=282)** | **Month 2**  **(n=278)** | **Month 3**  **(n=340)** | **Month 4**  **(n=268)** | **Month 5 (n=281)** |
| **Regular non-surgical site pain?**  Headache  Backache  Stomach pain  Other | 80 (28.4)  *36*  *31*  *17*  *20* | 55 (19.8)  *26*  *22*  *13*  9 | 64 (18.9)  *37*  *18*  *11*  *14* | 53 (19.8)  *27*  *18*  *12*  *11* | 47 (16.7)  *34*  *15*  *9*  *5* |
| **Trouble sleeping**  None  <25%  25-50%  51-75%  >75% | 187 (66.3)  53 (18.8)  21 (7.4)  13 (4.6)  8 (2.8) | 209 (75.2)  44 (15.8)  14 (5.0)  9 (3.2)  2 (0.7) | 261 (76.8)  47 (13.8)  19 (5.6)  9 (2.6)  4 (1.2) | 213 (79.5)  39 (14.6)  12 (4.5)  1 (0.4)  3 (1.1) | 223 (79.4)  38 (13.5)  14 (5.0)  3 (1.1)  3 (1.1) |
| **PHQ-9 score**†  0-4  5-9  10-14  15-19  20+ | 221 (78.4)  44 (15.6)  10 (3.5)  4 (1.4)  3 (1.1) |  | [n=278]  230 (82.7)  32 (11.5)  12 (4.3)  4 (1.4)  0 (0.0) |  | 237 (84.3)  34 (12.1)  4 (1.4)  4 (1.4)  2 (0.7) |
| **GAD-7 score**†**,** n%  0-4  5-9  10-14  15-21  **If problems, how difficult is life:**  Not difficult at all  Somewhat difficult  Very difficult  Extremely difficult | 240 (85.1)  26 (9.2)  11 (3.9)  5 (1.8)  86 (68.3)  29 (23.0)  8 (6.3)  3 (2.4) |  | [n=278]  228 (82.0)  30 (10.8)  17 (6.1)  3 (1.1)  73 (62.4)  33 (28.2)  8 (6.8)  3 (2.6) |  | 238 (84.7)  30 (10.7)  9 (3.2)  4 (1.4)  69 (62.7)  32 (29.1)  5 (4.5)  4 (3.6) |
| **Severe Pain Procedures only** | | | | | |
|  | **Month 1**  **(n=111)** | **Month 2**  **(n=108)** | **Month 3**  **(n=141)** | **Month 4**  **(n=106)** | **Month 5 (n=111)** |
| **Regular non-surgical site pain?**  Headache  Backache  Stomach pain  Other | 40 (36.0)  *8*  *21*  *6*  *12* | 26 (24.1)  *5*  *14*  *3*  *8* | 30 (21.4)  *11*  *11*  *3*  *8* | 24 (22.6)  *8*  *12*  *4*  *5* | 18 (16.2)  *11*  *7*  *2*  *3* |
| **Trouble sleeping**  None  <25%  25-50%  51-75%  >75% | 57 (51.4)  27 (24.3)  15 (13.5)  5 (4.5)  7 (6.3) | 71 (65.7)  22 (20.4)  9 (8.3)  4 (3.7)  2 (1.9) | 99 (70.2)  27 (19.1)  10 (7.1)  4 (2.8)  1 (0.7) | 79 (74.5)  20 (18.9)  6 (5.7)  0 (0.0)  1 (0.9) | 88 (79.3)  16 (14.4)  6 (5.4)  0 (0.0)  1 (0.9) |
| **PHQ-9 score**†  0-4  5-9  10-14  15-19  20+ | 81 (73.0)  21 (18.9)  4 (3.6)  3 (2.7)  2 (1.8) |  | [n=112]  89 (79.5)  14 (12.5)  8 (7.1)  1 (0.9)  0 (0.0) |  | 92 (82.9)  14 (12.6)  2 (1.8)  1 (0.9)  2 (1.8) |
| **GAD-7 score**†**,** n%  0-4  5-9  10-14  15-21  **If problems how difficult is life:**  Not difficult at all  Somewhat difficult  Very difficult  Extremely difficult | 92 (82.9)  14 (12.6)  4 (3.6)  1 (0.9)  [n=60]  36 (60.0)  17 (28.3)  5 (8.3)  2 (3.3) |  | [n=112]  87 (77.7)  14 (12.5)  10 (8.9)  1 (0.9)  [n=57]  32 (56.1)  19 (33.3)  4 (7.0)  2 (3.5) |  | 90 (81.1)  13 (11.7)  5 (4.5)  3 (2.7)  [n=49]  31 (63.3)  14 (28.6)  2 (4.1)  2 (4.1) |
| †Abbreviations: Patient Health Questionnaire-9 (PHQ-9; screening tool for depression); Generalized Anxiety Disorder-7 (GAD-7; screening tool for anxiety) | | | | | |
